# The Encoding of Temporally Irregular and Regular Visual Patterns in the Human Brain

**DOI:** 10.1371/journal.pone.0002180

**Published:** 2008-05-14

**Authors:** Semir Zeki, Oliver J. Hulme, Barrie Roulston, Michael Atiyah

**Affiliations:** 1 Wellcome Laboratory of Neurobiology, Anatomy Department, University College London, London, United Kingdom; 2 School of Mathematics, University of Edinburgh, Edinburgh, United Kingdom; University of Southern California, United States of America

## Abstract

In the work reported here, we set out to study the neural systems that detect predictable temporal patterns and departures from them. We used functional magnetic resonance imaging (fMRI) to locate activity in the brains of subjects when they viewed temporally regular and irregular patterns produced by letters, numbers, colors and luminance. Activity induced by irregular sequences was located within dorsolateral prefrontal cortex, including an area that was responsive to irregular patterns regardless of the type of visual stimuli producing them. Conversely, temporally regular arrangements resulted in activity in the right frontal lobe (medial frontal gyrus), in the left orbito-frontal cortex and in the left pallidum. The results show that there is an abstractive system in the brain for detecting temporal irregularity, regardless of the source producing it.

## Introduction

A pattern may be defined as an arrangement or sequence that follows, or is reducible to, some rule or principle, and is characterized by some regularity in the relations among its constituents. This contrasts with arrangements that are irregular, and in which consequently no rule or principle can be discerned. The capacity to detect the presence or absence of regularity allows us to simplify or reduce sets of observations to their underlying rules and laws. Indeed, all sciences search for some aspect of regularity or patterning and nowhere is this truer than in mathematics, which can even be said to be the science of patterns.

Regularities and irregularities that can be detected by sense organs are the most relevant to the brains of living organisms, as well as being the easiest to study. In the work reported here, we addressed the problem of whether the brain has any system for registering sequences of visual stimuli in terms of their temporal regularity and irregularity. Given that abstraction is such a ubiquitous feature of cerebral physiology [1], we hypothesised that there would be a hierarchical pattern-sensitive system which shows different levels of invariance to patterns. Since evaluating the regularity of a pattern in a sequence of stimuli would almost certainly involve working memory, we also hypothesised that this system would be dependent on prefrontal regions.

## Materials and Methods

### Subjects

Sixteen healthy, right-handed volunteers acted as subjects (seven females, mean age 26.4 yrs; nine males, mean age 29.9 yrs). All gave informed consent, and the Ethics Committee of the National Hospital for Neurology and Neurosurgery, London, UK granted ethical approval for the study.

### Stimuli

All stimuli were created using Cogent 2000 Graphics (available at www.vislab.ucl.ac.uk/Cogent) running in MATLAB (Mathworks Inc). They consisted of temporally regular or irregular sequences in four visual submodalities: Color, Luminance, Letters and Numbers, thus leading to eight experimental conditions of interest (2×4 factorial design). In the former, a set of three elements (eg three different colors or letters) belonging to a visual submodality (e.g. color or numbers) appeared with a regular order of repeating triplets, whereas in the latter they appeared in an irregular, non-repeating, order (in which the same element never appeared twice in immediate succession).

### Procedure

Between one day and one week before the subjects were due to be scanned, they were trained on the task that they would perform in the scanner; this also allowed us to measure their performance and thus compare task difficulty for the different conditions.

**Figure 1:**
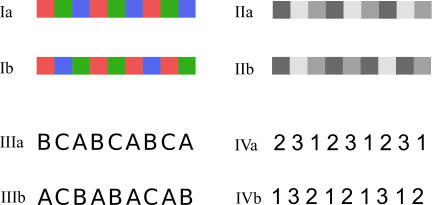
Examples of the stimuli used in this study. Time runs from left to right with the upper row of each box depicting a regular sequence and the lower row an irregular one. I) color II) luminance III) letters IV) numbers.

A single trial consisted of nine elements of a single visual submodality only, appearing in either a regular or in an unpredictable temporal sequence, as shown in Figure 1. Each trial of nine elements lasted 4.5 s (0.5 s for each element) and there was a 1.3 s pause between trials. Throughout, subjects were asked to respond by pressing a button with their right index finger when they perceived a regular temporal pattern and the right middle finger when they perceived an irregular one.

A block of trials consisted of four regular trials and four irregular ones in a randomised sequence, each of the same submodality (either Color, Luminance, Letters or Numbers). There was an extra period of duration 2.5 s between each block. A session of blocks included a representative block of each of the four visual submodalities in a randomised sequence and the experiment for each subject consisted of three complete sessions. For the color and luminance conditions, the stimuli subtended 6.3°×6.3° while letters and ciphers had a vertical size of 5°. Colored stimuli were made equiluminant using the technique of heterochromatic flicker photometry [2].

### Scanning Details

Subjects were scanned in a 1.5 T Siemens Sonata scanner with a head volume coil. A gradient EPI sequence was used (TE = 90 ms, repeat time 3.42 s). Each brain image was acquired in a descending sequence comprising 38 slices, each 2 mm thick with 1 mm gaps in-between, and consisting of 64×64 voxels. A total of 360 acquisitions was made. Each subject was scanned again after the functional scanning session with a T1-weighted structural sequence.

### Data Analysis

Data were analysed using the SPM2 software (www.fil.ion.ucl.ac.uk). The echo planar images were spatially realigned to the first image and then re-sliced to produce a voxel size of 2×2×2 mm. All images were spatially normalized to the Montreal Neurological Institute (MNI) template provided in SPM2. They were then spatially smoothed with a Gaussian kernel of 14 mm full width at half maximum. The resulting images were temporally filtered using a high-pass filter with a low frequency cut off of 1/400 Hz to remove slowly varying scanner noise.

The experiment was an event-related design in which each event was convolved with a canonical haemodynamic response function (HRF) and then entered into a multiple linear regression model. The analyses described below were all performed at the random effects level so that the reliability of measurements could be assessed in relation to the between subject variance [3]. Each of the eight trial types was modelled separately in the design matrix and treated as effects of interest. The head movement parameters obtained whilst realigning each image, and the subjects' button presses, were included in the analysis as effects of no interest.

The resultant parameter estimates for each regressor (at each voxel) were compared using *t* tests to determine whether there were significant differences in activation between conditions. The statistical results given are based on a single-voxel *t* statistic corresponding to *p*<0.001, uncorrected for multiple comparisons (unless otherwise stated). The co-ordinates of all activations are reported in MNI space.

## Results

### Scanning data

#### A. The effects of temporal sequences on activity in specialized visual areas

Given the functional specialization of the visual brain, with different visual submodalities being processed in distinct visual areas [4], we wanted to show, first, that stimulation with a given submodality (e.g. color) activates the area specialized for that submodality (in this instance V4). To do so, we first localized the specialized areas in this study, by contrasting the effects produced by a stimulus belonging to one submodality against the effects produced by the other three. (a) In the contrast Numbers vs. others, the main activity was in the right hemisphere at 40, -94, -4 while (b) in that of Letters vs. others, it was in left hemisphere at –40, -90, -4. These co-ordinates correspond to the lateral occipital complex (LOC), a large cortical zone specialized for shape and object perception and in which several subdivisions have been recognized [5], [6]. (c) The contrast Luminance vs. others activated the right occipital lobe at 26, -66, -12 and the right frontal lobe at 46, 44, 4 while d) the contrast Color vs. all other conditions activated area V4, specialized for the processing and perception of color [7], [8], [9]. Here the activation was bilateral but more significant in the right hemisphere (at –12, -94, 18; p<0.05 corrected). Hence stimuli belonging to a given visual submodality produced, as predicted, activity in a correspondingly specialized area of the visual brain. We then determined whether the activity produced in a specialized area is modulated according to whether the visual stimulus for which the area is specialized is presented in a regular or an unpredictable sequence, by studying the contrast (regular vs irregular) and its converse for each submodality. The results showed that there was no significant effect, even at liberal thresholds, of either regularity or irregularity on the activity in any of the specialized, sub-modality specific, areas themselves.

#### B. Main effects of irregularity and regularity

**Figure 2:**
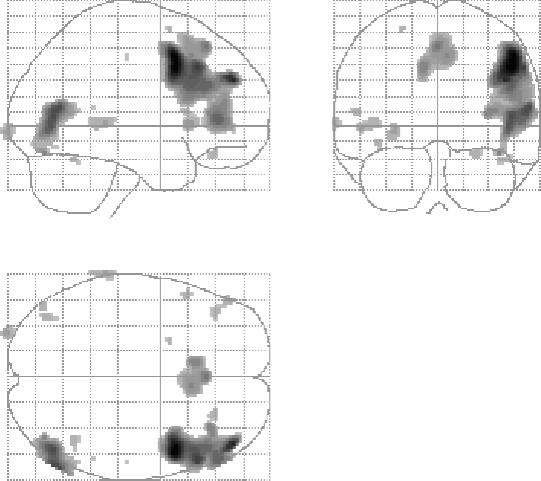
Glass brain projections to show the areas of activation produced by the comparison of Irregular vs. Regular sequences.

Our next step was to determine whether the presentation of stimuli in regular or irregular temporal order, regardless of the submodality (i.e. whether they were color or luminance stimuli) led to different activation patterns in the brain. The main effects of temporal irregularity, obtained through the contrast Temporal Irregular vs. Temporal Regular (for all submodalities), showed broad activity in the dorsolateral frontal cortex, corresponding to, but extending beyond, Brodmann Area 46 (p<0.001 uncorrected) into Brodmann areas 9, 45 and 44. The same contrast also revealed activity in the occipital lobe, at 58, -68, 10, in a region corresponding to the LOC (Figure 2).

**Figure 3:**
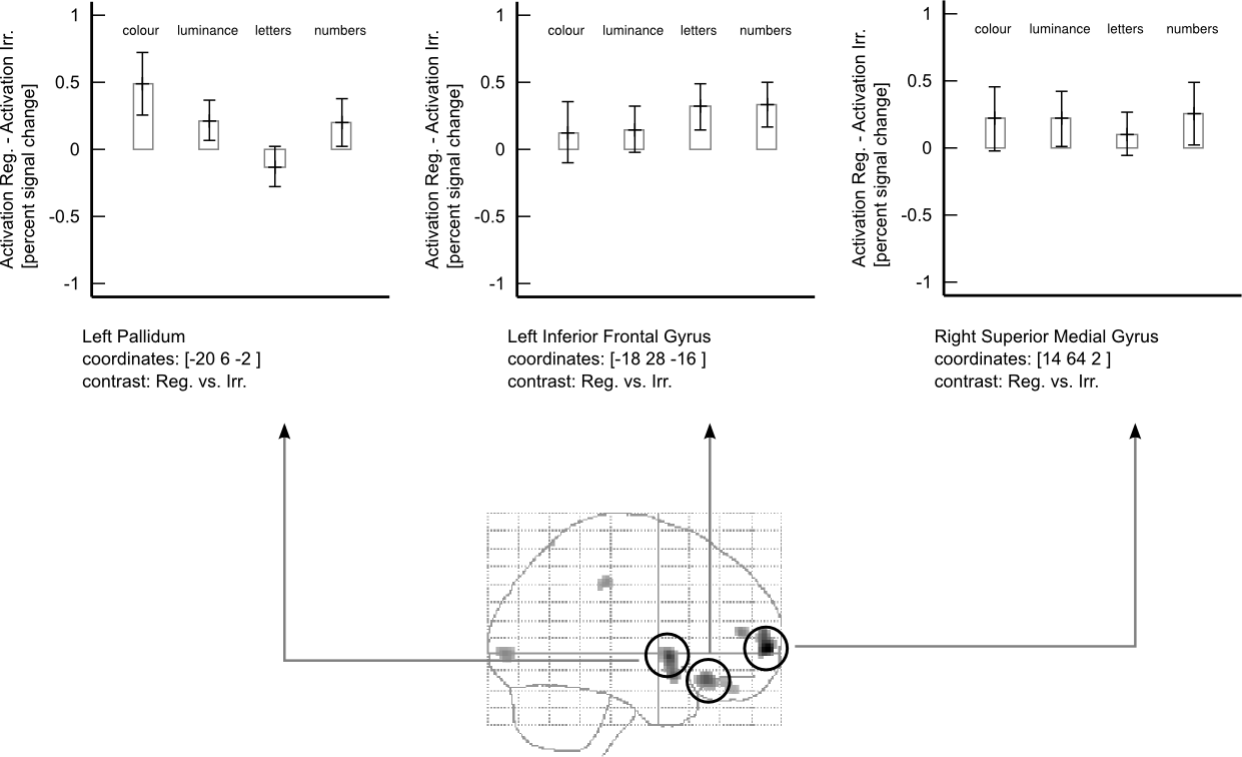
Brain activity shown as glass-brain projections for the contrasts Regular vs. Irregular, with parameter estimates for the hottest voxel of each area of activation shown above.

The main effect of temporal regularity, obtained with the contrast Regular vs. Irregular (for all submodalities), showed activity in (i) in the right frontal lobe, at 14, 64, 2; (ii) in the left pallidum at -20, 6, -2, (iii) in the left orbito-frontal cortex at -18, 28, -16, with parameter estimates (Figure 3) showing that each stimulus modality had a significant effect.

#### C. Conjunction Analyses across visual submodalities

**Figure 4:**
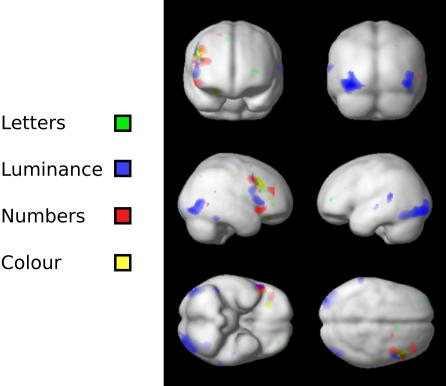
Brain areas activated in the comparison Irregular vs. Regular, to show the area of confluence of activity in the frontal lobe of the right hemisphere (right).

**Figure 5:**
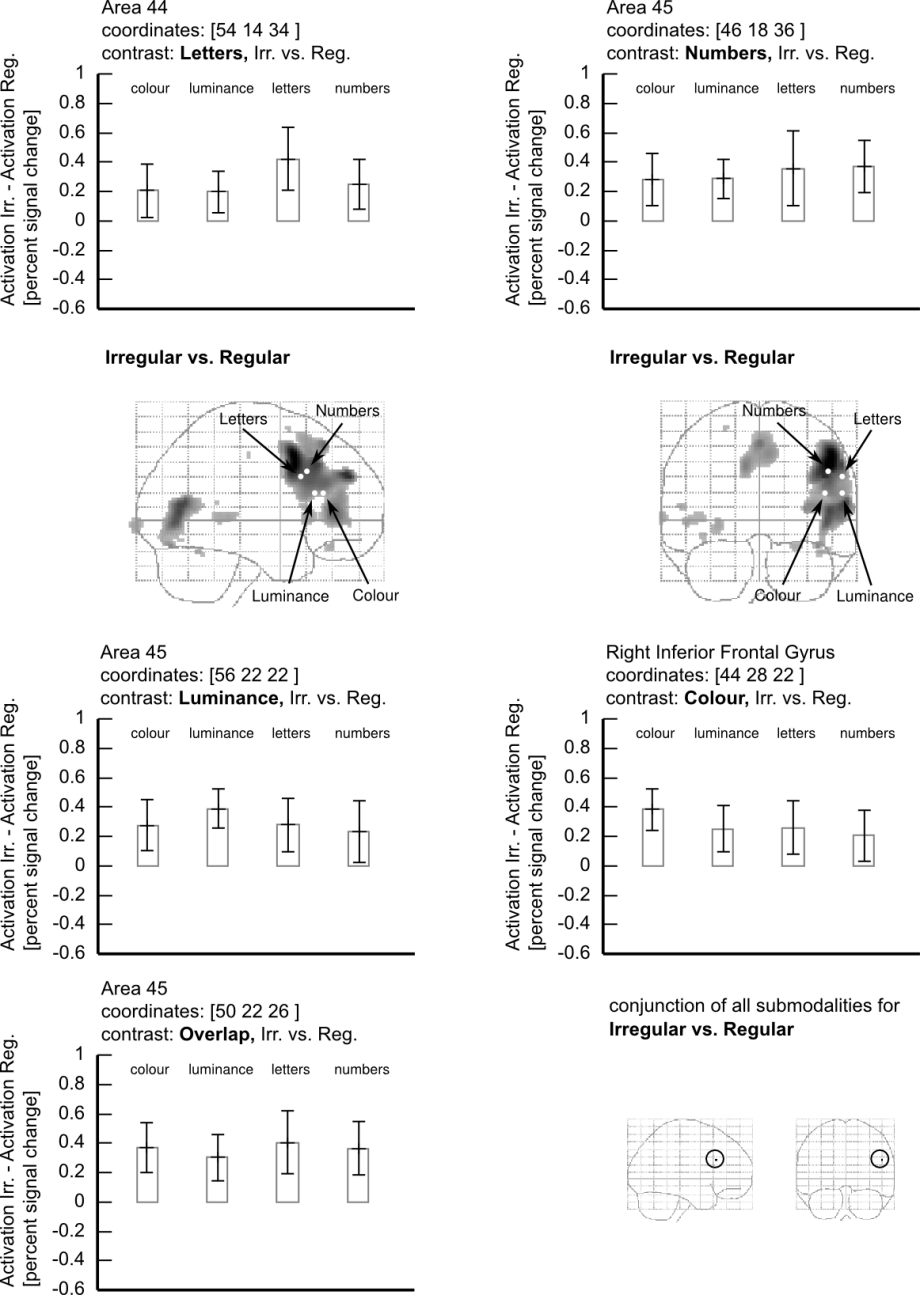
Plots to show the difference in parameter estimates for the hottest voxel in the contrast of Irregular vs Regular obtained for each submodality. The lower left panel shows the same for the region of overlap as identified by a conjunction of all 4 contrasts, and the lower right panel is a glass-brain projection to show the region of overlap. The equivalent conjunction analysis of Regular vs. Irregular did not show any common area to be active. All effects shown here are significant at p<0.001, uncorrected.

*(i) Irregular vs. Regular*: The largest activations were produced by the contrast Irregular vs. Regular for each submodality. Each produced activity in the dorsolateral prefrontal cortex, with different foci for each distributed within the cortical zone found in the main effect for the contrast Irregular vs. Regular. We wanted to learn whether there was a common area in the dorsolateral prefrontal cortex which was activated whenever a temporally irregular visual pattern was presented, regardless of the submodality used to generate it. A conjunction analysis of the form: {Irregular *vs.* Regular} across all four submodalities revealed a peak voxel activation at 50, 22, 26 in the right dorsolateral prefrontal cortex. **Figure 4** shows that the common area activated in the above contrast is at the confluence of the three areas activated by the individual contrasts of Irregular vs. Regular. We conclude that this region responds to irregular sequences regardless of the submodality in which they are defined. **Figure 5** shows the parameter estimates for each of the areas active in the irregular vs regular contrasts, as well as for the common area that was active with irregularity regardless of its origin.

*(ii) Regular vs. Irregular:* We performed another conjunction analysis of the form {Regular vs. Irregular} across all submodalities, to determine whether there was any area in which there was an overlap of activity between Regular vs. Irregular for all visual submodalities. The comparison did not find any area that was conjointly active in this contrast for all 4 submodalities (p<0.001 uncorrected).

### Behavioral data

There were remarkably few significant differences in the mean response times for the regular and irregular stimuli for the majority of subjects. Only subjects 1, 4, 5 and 12 showed a significant difference (p<0.05 corrected) for 1 or more submodality (see Table S1). When such subjects were excluded from the analysis, all of the imaging results reported (the inferences and associated significance levels) remained the same. Comparing the percentage correct scores for each condition, there were no significant differences between performance for regular vs. irregular conditions for the intensity, letters and numbers condition. For the color condition however performance scores were significantly higher (p<0.05 corrected) for the irregular stimuli.

## Discussion

We manipulated the regularity of the temporal sequences of visual stimuli for four different visual submodalities and studied their effects on brain activity. The results suggest the existence of an abstractive process in registering temporal irregularities. In all cases modality specific regions were found in prefrontal cortex, centred on BA 46 but extending beyond, which responded more to irregular than regular sequences. There was, however, a region within this network that showed greater activity for irregular versus regular sequences for all visual submodalities. There would thus seem to be specialized systems for processing irregular sequences, consisting of modules that are specialised for the submodality in which the sequence is expressed. In addition, there would seem to be a submodality invariant region, whose response is abstract in the sense that any type of temporal irregular arrangement can drive it. This implies a hierarchy in the abstractive process, with specialized frontal areas abstracting the temporally irregular pattern within a specific submodality. At a higher level in the hierarchy is another area, which is only concerned with irregular temporal patterns, without being concerned with the particular submodality in which the pattern is expressed.

There was no such finding in the Regular vs. Irregular sequence contrasts, nor was there any apparent confluence of submodality specific regularity sensitive areas. Our results could be taken to suggest that the kind of abstractive hierarchical system used in the detection of temporal irregularity is not used in the detection of regularity. This is not to say that abstraction is not occurring for these stimuli, since these areas responded irrespective of the precise stimulus configurations. In other words, it made little difference which color succeeded which in regular sequence, or which precise luminance values succeeded each other. The results therefore show rather that the abstraction is not hierarchically organised across different visual submodalities as is the system for registering irregularities.

### The prefrontal cortex and expectation

That the strongest activation should have been with irregular, and therefore unpredictable, sequences and that these should have activated the frontal cortex centred on BA 46 is important. The frontal cortex has been found to be active with “oddball” visual and auditory tasks, that is for conditions in which expectations are violated [10], [11], [12], [13]. In these previous experiments, an unexpected target (e.g. XXXX) was presented infrequently amidst the much more frequent target (e.g. OOOO). Equally, presentation of objects in unexpected colors results in activity in BA 46 [14]. In our experiment, subjects simply viewed (followed by a button press) regular or irregular sequences belonging to the same sub-modality, for example color. Given that there was also activity in frontal cortex, though a different subdivision of it, in response to regular patterns, it would seem that the brain is equipped to register both departures from and confirmations of regularity.

### Prefrontal cortex and temporal processing

That the activity specific to sequences is expressed solely in prefrontal cortex is especially interesting when considered in light of current theories of prefrontal function. One account suggests that the lateral prefrontal cortex underlies the temporal organisation of behaviour [15], by integrating information across time: prefrontal mechanisms are involved when there is a temporal discontinuity in a sensory sequence. For instance, electrophysiological studies have shown macaque prefrontal neurons to respond to task-relevant sensory associations across time [16], [17]. In addition, human neuroimaging studies have shown that the right dorsolateral prefrontal cortex is activated by behavioural responses contingent on temporal context [18], and unpredictable sensory events across time [e.g. 19] in a way that is predictive of subsequent behavioural changes.

Overall, our findings can be summarised as follows: 1) The dorsolateral prefrontal cortex contains a confluence of regions each selective for the temporal irregularity of different visual submodalities. 2) Within this confluence of regions there is a region sensitive to temporal irregularity that is invariant to the submodality from which it is defined. 3) For temporally regular patterns, disparate regions are recruited with no common area that responds invariantly across visual submodalities.

## Supporting Information

Table S1 Supplementary data documenting response scores and response times for reacting to the patterns generated from the different submodalities.
(0.08 MB DOC)
