# Supplementary material for: The Encoding of Temporally Irregular and Regular Visual Patterns in the Human Brain
Source: PLoS One. 2008 May 14;3(5):e2180. doi: 10.1371/journal.pone.0002180 (PMC2364658; doi:10.1371/journal.pone.0002180)
Supplement: Table S1 — Supplementary data documenting response scores and response times for reacting to the patterns generated from the different submodalities. (0.08 MB DOC) [file pone.0002180.s001.doc]

### SUPPLEMENTARY DATA

The tables below give scores and response times for all sixteen subjects for the four submodalities Colour, Brightness, Letters and Numbers. Each submodality has a column for the Irregular sequences and one for the Regular sequences.

|  | Irregular Colour | | | Regular Colour | | | Irregular Brightness | | | Regular Brightness | | |
| --- | --- | --- | --- | --- | --- | --- | --- | --- | --- | --- | --- | --- |
| ID | Score | Mean | SSD | Score | Mean | SSD | Score | Mean | SSD | Score | Mean | SSD |
| 1 | 12/12 | 2.820 | 0.543 | 11/12 | 3.569 | 0.563 | 10/12 | 3.627 | 0.683 | 11/12 | 4.319 | 0.670 |
| 2 | 12/12 | 3.310 | 0.688 | 11/12 | 3.620 | 0.703 | 7/9 | 4.294 | 0.813 | 11/12 | 3.757 | 0.924 |
| 3 | 12/12 | 2.958 | 0.482 | 12/12 | 3.355 | 0.482 | 11/12 | 4.049 | 0.597 | 11/12 | 3.694 | 0.753 |
| 4 | 12/12 | **3.722** | **0.772** | 12/12 | **4.998** | **0.321** | 9/11 | 4.405 | 1.029 | 8/9 | 5.287 | 0.318 |
| 5 | 12/12 | 3.480 | 0.838 | 11/12 | 4.322 | 0.573 | 12/12 | 4.080 | 0.873 | 11/12 | 4.007 | 0.624 |
| 6 | 12/12 | 3.738 | 0.968 | 11/11 | 3.468 | 0.777 | 9/11 | 4.155 | 0.787 | 11/12 | 3.742 | 0.731 |
| 7 | 12/12 | 4.598 | 0.439 | 8/12 | 4.205 | 0.954 | 12/12 | 4.880 | 0.527 | 10/12 | 4.461 | 0.734 |
| 8 | 12/12 | 4.439 | 0.964 | 10/11 | 4.407 | 0.708 | 12/12 | 4.524 | 0.613 | 12/12 | 4.191 | 0.427 |
| 9 | 10/12 | 3.388 | 0.669 | 10/12 | 3.551 | 0.661 | 9/12 | 3.637 | 0.497 | 12/12 | 3.994 | 0.661 |
| 10 | 12/12 | 4.138 | 0.616 | 11/12 | 4.568 | 0.332 | 8/12 | 4.192 | 0.638 | 6/11 | 4.877 | 0.432 |
| 11 | 8/10 | 4.153 | 0.828 | 10/11 | 4.525 | 1.074 | 10/11 | 5.074 | 0.546 | 6/9 | 5.225 | 0.447 |
| 12 | 12/12 | **3.179** | **0.617** | 12/12 | **4.312** | **0.110** | 10/12 | 4.370 | 0.740 | 12/12 | 4.441 | 0.230 |
| 13 | 12/12 | 3.839 | 1.036 | 10/11 | 4.770 | 0.470 | 12/12 | 4.712 | 0.824 | 11/12 | 4.620 | 0.508 |
| 14 | 12/12 | 3.751 | 0.810 | 12/12 | 3.505 | 0.763 | 10/10 | 4.875 | 0.517 | 9/9 | 4.167 | 0.614 |
| 15 | 12/12 | 5.242 | 0.254 | 8/9 | 5.228 | 0.287 | 9/10 | 5.236 | 0.206 | 10/10 | 5.032 | 0.242 |
| 16 | 12/12 | 5.032 | 0.491 | 11/12 | 4.899 | 0.260 | 11/11 | 5.170 | 0.203 | 12/12 | 4.812 | 0.284 |

|  | Irregular Letters | | | Regular Letters | | | Irregular Numbers | | | Regular Numbers | | |
| --- | --- | --- | --- | --- | --- | --- | --- | --- | --- | --- | --- | --- |
| ID | Score | Mean | SSD | Score | Mean | SSD | Score | Mean | SSD | Score | Mean | SSD |
| 1 | 12/12 | 3.431 | 0.825 | 12/12 | 3.775 | 0.795 | 12/12 | **2.819** | **0.351** | 12/12 | **3.696** | **0.627** |
| 2 | 12/12 | 3.524 | 0.851 | 11/12 | 3.208 | 0.367 | 9/12 | 3.238 | 0.667 | 11/11 | 3.140 | 0.575 |
| 3 | 12/12 | 3.337 | 0.377 | 12/12 | 3.490 | 0.397 | 12/12 | 3.221 | 0.438 | 12/12 | 3.322 | 0.585 |
| 4 | 11/11 | 3.716 | 0.819 | 10/10 | 4.778 | 0.602 | 11/11 | **3.590** | **0.591** | 11/11 | **4.792** | **0.204** |
| 5 | 11/12 | **3.194** | **0.669** | 11/12 | **4.442** | **0.416** | 11/12 | 3.584 | 1.115 | 12/12 | 4.410 | 0.311 |
| 6 | 12/12 | 3.060 | 0.597 | 12/12 | 3.344 | 0.594 | 9/11 | 3.313 | 0.746 | 11/12 | 3.625 | 0.685 |
| 7 | 11/12 | 4.230 | 0.689 | 12/12 | 3.473 | 0.608 | 11/12 | 4.011 | 0.780 | 12/12 | 4.193 | 0.852 |
| 8 | 12/12 | 4.267 | 0.908 | 12/12 | 3.891 | 0.696 | 12/12 | 4.333 | 0.557 | 11/11 | 3.858 | 0.763 |
| 9 | 12/12 | 3.227 | 0.451 | 12/12 | 3.522 | 0.793 | 11/12 | 3.382 | 0.705 | 12/12 | 3.323 | 0.324 |
| 10 | 12/12 | 3.803 | 0.818 | 10/12 | 4.522 | 0.500 | 11/12 | 3.952 | 0.850 | 11/12 | 4.136 | 0.550 |
| 11 | 12/12 | 4.096 | 1.550 | 10/10 | 4.095 | 0.936 | 11/11 | 4.755 | 0.893 | 11/12 | 4.909 | 0.667 |
| 12 | 12/12 | **2.811** | **0.387** | 12/12 | **4.202** | **0.089** | 12/12 | **2.918** | **0.731** | 12/12 | **4.016** | **0.205** |
| 13 | 12/12 | 4.366 | 0.733 | 10/11 | 4.557 | 0.702 | 12/12 | 4.577 | 0.837 | 12/12 | 5.103 | 0.557 |
| 14 | 12/12 | 3.650 | 0.939 | 12/12 | 3.303 | 0.728 | 11/12 | 3.948 | 0.922 | 12/12 | 3.639 | 0.666 |
| 15 | 11/12 | 5.135 | 0.339 | 8/10 | 5.168 | 0.297 | 10/12 | 5.168 | 0.563 | 11/12 | 5.136 | 0.311 |
| 16 | 12/12 | 5.018 | 0.225 | 10/11 | 4.911 | 0.359 | 11/12 | 5.106 | 0.221 | 10/10 | 4.818 | 0.149 |

Scores refer to the number of times the subject correctly identified the sequence type (irregular/regular). Although there were twelve trials in each case the subject may not have made a response in each case so for example the score of 8/10 for subject 11 in the “Irregular Colour” column indicates that they responded “Irregular” eight times, “Regular” twice and on another two occasions they made no response. The Mean and SSD columns give the mean response time in seconds and the sample standard deviation.

**Response Times**

We can use response time as an indicator of the complexity of the task to see whether there is a significant difference between the response times for the irregular and regular sequences. However, it should be borne in mind that there is an inherent flaw in using the response time because an irregular sequence becomes apparent at the onset of the first irregular element in the sequence whereas a regular sequence can only be confirmed after all the elements have been revealed. So we might predict that the irregular sequence response times would be shorter than the regular sequence response times.

For each submodality and each subject a two-sample, two-sided t-test is performed (H_0_: μ_Irregular_ = μ_Regular_, H_1_: μ_Irregular_ ≠ μ_Regular_). A bold entry in the tables above indicates that the null hypothesis can be rejected at the p < 0.05 level (corrected for 64 multiple comparisons).

There are remarkably few significant differences in the response times for the regular and irregular stimuli for the majority of subjects; only subjects 1, 4, 5 and 12 ever show a significant difference and in these cases it is invariably that the irregular response time is shorter than the regular (as we might expect).

We can also compare the mean response times for regular and irregular stimuli across all subjects using a sign test and the mean response time for each subject (again H_0_: μ_Irregular_ = μ_Regular_, H_1_: μ_Irregular_ ≠ μ_Regular_). In all cases the sign test gives a statistically insignificant (p > 0.05 uncorrected) split of 6:10 for all 4 modes (for Colour, Letters and Numbers Irr. < Reg. but for Intensity Reg > Irr.).

**Scores**

Finally we can perform a similar sign test on the irregular and regular scores for each subject and for each submodality. The scores should give a more accurate indication of task complexity because the precise time that the sequence can be correctly identified should not affect the correctness of the response. Here a missing response has been counted as an incorrect response (i.e. a score of 8/10 becomes 8/12). In these tests the colour scores were significantly higher for the irregular sequences than for the regular sequences (at the p < 0.05 level even when corrected for 4 comparisons). None of the other score comparisons (for intensity, letters and numbers) was significant (p > 0.05, uncorrected).
